# Food variety, dietary diversity, and type 2 diabetes in a multi-center cross-sectional study among Ghanaian migrants in Europe and their compatriots in Ghana: the RODAM study

**DOI:** 10.1007/s00394-017-1538-4

**Published:** 2017-09-25

**Authors:** Ina Danquah, Cecilia Galbete, Karlijn Meeks, Mary Nicolaou, Kerstin Klipstein-Grobusch, Juliet Addo, Ama de-Graft Aikins, Stephen K. Amoah, Peter Agyei-Baffour, Daniel Boateng, George Bedu-Addo, Joachim Spranger, Liam Smeeth, Ellis Owusu-Dabo, Charles Agyemang, Frank P. Mockenhaupt, Erik Beune, Matthias B. Schulze

**Affiliations:** 1 Department of Molecular Epidemiology, German Institute of Human Nutrition Potsdam-Rehbruecke (DIfE), Arthur-Scheunert-Allee 114-116, 14558 Nuthetal, Germany; 2 Institute for Social Medicine, Epidemiology and Health Economics, Charité-Universitaetsmedizin Berlin, Corporate Member of Freie Universität Berlin, Humboldt-Universität zu Berlin, and Berlin Institute of Health, Berlin, Germany; 3 Department of Public Health, Academic Medical Center, University of Amsterdam, Amsterdam, The Netherlands; 4 Julius Global Health, Julius Center for Health Sciences and Primary Care, University Medical Center Utrecht, Utrecht, The Netherlands; 5 Division of Epidemiology and Biostatistics, School of Public Health, Faculty of Health Sciences, University of the Witwatersrand, Johannesburg, South Africa; 6 Department of Non-communicable Disease Epidemiology, Faculty of Epidemiology and Population Health, London School of Hygiene and Tropical Medicine, London, UK; 7 Regional Institute for Population Studies, University of Ghana, Legon-Accra, Ghana; 8 Institute of Tropical Medicine and International Health, Charité-Universitaetsmedizin Berlin, Corporate Member of Freie Universitaet Berlin and Humboldt-Universitaet zu Berlin, and Berlin Institute of Health, Berlin, Germany; 9 Faculty of Science, Kwame Nkrumah University of Science and Technology, Kumasi, Ghana; 10 School of Medical Sciences, Kwame Nkrumah University of Science and Technology, Kumasi, Ghana; 11 Department of Endocrinology and Metabolism, DZHK (German Centre for Cardiovascular Research), Partner Site Berlin, Center for Cardiovascular Research (CCR), Charité-Universitaetsmedizin Berlin, Corporate Member of Freie Universitaet Berlin and Humboldt-Universitaet zu Berlin, and Berlin Institute of Health, Berlin, Germany

**Keywords:** Food variety, Dietary diversity, Dietary patterns, Type 2 diabetes, Africa

## Abstract

**Purpose:**

The importance of dietary diversification for type 2 diabetes (T2D) risk remains controversial. We investigated associations of between- and within-food group variety with T2D, and the role of dietary diversification for the relationships between previously identified dietary patterns (DPs) and T2D among Ghanaian adults.

**Methods:**

In the multi-center cross-sectional Research on Obesity and Diabetes among African Migrants (RODAM) Study (*n* = 3810; Ghanaian residence, 56%; mean age, 46.2 years; women, 63%), we constructed the Food Variety Score (FVS; 0–20 points), the Dietary Diversity Score (DDS; 0–7 points), and the Diet Quality Index-International (DQI-I) variety component (0–20 points). The associations of these scores, of a “rice, pasta, meat and fish” DP, of a “mixed” DP, and of a “roots, tubers and plantain” DP with T2D were calculated by logistic regression.

**Results:**

The FVS was inversely associated with T2D, adjusted for socio-demographic, lifestyle, and anthropometric factors [odds ratio (OR) for T2D per 1 standard deviation (SD) increase: 0.81; 95% confidence interval (CI) 0.71–0.93]. The DDS and the DQI-I variety component were not associated with T2D. There was no association of the “mixed” DP and the “roots, tubers and plantain” DP with T2D. Yet, the “rice, pasta, meat and fish” DP is inversely associated with T2D (OR for T2D per 1 SD increase: 0.82; 95% CI 0.71–0.95); this effect was slightly attenuated by the FVS.

**Conclusions:**

In this Ghanaian population, between-food group variety may exert beneficial effects on glucose metabolism and partially explains the inverse association of the “rice, pasta, meat and fish” DP with T2D.

**Electronic supplementary material:**

The online version of this article (doi:10.1007/s00394-017-1538-4) contains supplementary material, which is available to authorized users.

## Introduction

Type 2 diabetes (T2D) constitutes a major public health challenge among sub-Saharan African populations, both in the countries of origin and for African minority populations in Europe. For Ghanaian adults, the prevalence of T2D is 5% in rural Ghana, 10% in urban Ghana, and 8–15% in Europe [1]. Dietary behavior is an important modifiable risk factor for T2D [2], and dietary pattern analysis facilitates the investigation of this complex lifestyle factor [3]. Dietary diversification has been propagated as a health-beneficial component of dietary behavior [2], and complements the concept of exploratory dietary patterns (DPs) [4]. In low- and middle-income countries (LMICs), dietary diversification has been examined, primarily concerning malnutrition-related health outcomes [5, 6]. Regarding metabolic conditions, within-food group variety showed health-beneficial effects in some LMICs, such as Benin and Malaysia [7, 8], while this was not uniformly observed and was absent for between-food group variety [9–11]. Also, among ethnic minority populations in high-income countries (HICs), data regarding dietary diversification and the risk of T2D remain scarce and so far inconclusive [12–14]. For instance, Tunisian migrants in France showed better between- and within-food group varieties than French natives, which partly explained the lower prevalence of T2D in this African migrant population [13]. This was not seen among African Americans in the large Coronary Artery Risk Development in Young Adults (CARDIA) study [14].

Previously, we have established exploratory DPs among adult Ghanaians residing in Ghana and Europe [15]. Still, the importance of dietary diversification and of these identified DPs for T2D risk remain to be examined in this population. Therefore, the aim of the present study was to investigate the associations of scores reflecting dietary diversification and previously identified DPs with T2D. Further, we aimed at examining the contributions of dietary diversification to the DPs-T2D relationships.

## Methods

### Study design and study population

**Fig. 1:**
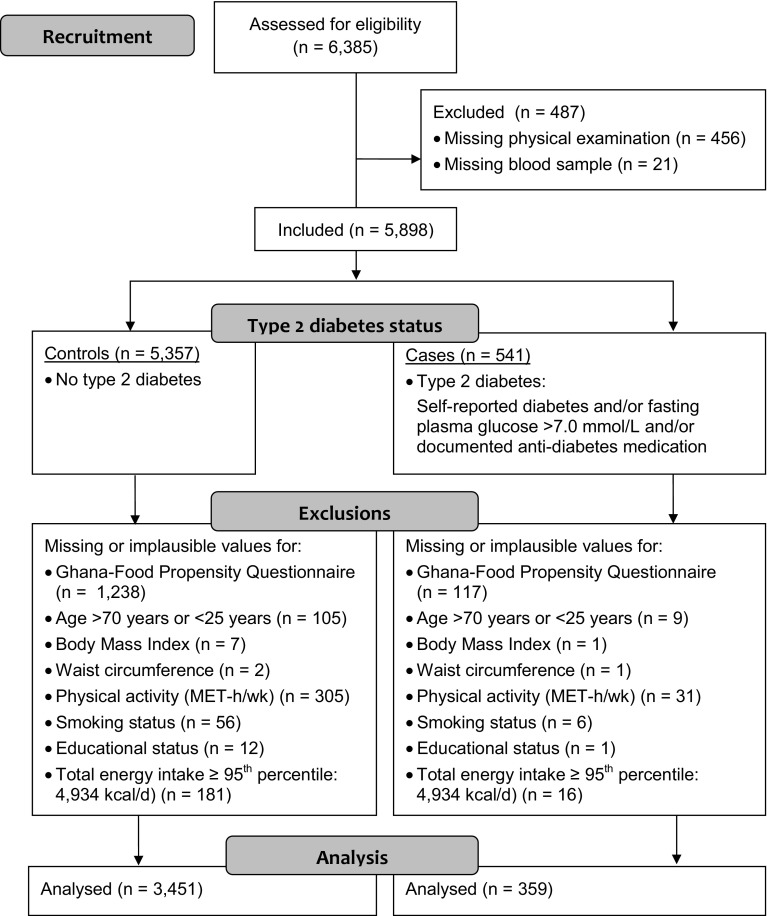
Flow diagram of excluded participants

The study protocol and procedures of the Research on Obesity and Diabetes among African Migrants (RODAM) study have been published elsewhere [16]. In brief, this multi-center cross-sectional study was conducted among Ghanaian adults (25–70 years) in rural Ghana, urban Ghana, and Europe (Amsterdam, London, and Berlin) between July 2012 and September 2015 (*n* = 6385). The primary objective of the RODAM study was to disentangle the relative contribution of (epi) genetic and non-genetic risk factors for obesity and T2D. For recruitment, in Ghana, census data of 2010 were used to draw rural and urban participants in the Ashanti region. In Amsterdam, the Municipal Health Register was used to randomly select Ghanaian migrants who have been invited by postal mail and home visits. In London and Berlin, Ghanaian organizations, church communities, and social unions served as the sampling frame for recruitment. The response rates were 76% in rural Ghana and 74% in urban Ghana. In Amsterdam, 67% replied by response card or after a home visit. Of these, 53% agreed and participated in the study. In London, of those individuals who were invited based on their registration in Ghanaian organizations, 75% agreed and participated in the study. In Berlin, this figure was 68%. For the present analysis, Fig. 1 presents the flow chart of excluded participants because of missing or implausible data, resulting in a final analytical sample size of 3810 participants.

All blood samples were collected, handled, processed, and stored according to standardized procedures and were analyzed in the same laboratory in Berlin (Charité) to avoid variability between laboratories. Fasting plasma glucose (FPG; mmol/L) was measured in fasting venous blood (ABX Pentra 400 chemistry analyzer; HORIBA ABX SAS, Montpellier, France). T2D was defined as FPG ≥ 7.0 mmol/L or documented glucose-lowering medication or self-reported diabetes. Trained study personnel performed the dietary assessment and the anthropometric measurements, according to standardized operating procedures across all study sites. Medical history, lifestyle, and socio-economic factors were recorded either in questionnaire-based interviews or by self-report.

### Dietary assessment

Details of the dietary assessment have previously been described [15]. In brief, a semi-quantitative Ghana-Specific Food Propensity Questionnaire (Ghana-FPQ) was developed to assess the usual dietary intake of 134 food items. The Ghana-FPQ queries for the intake frequencies of food groups at pre-defined portion sizes in the preceding 12 months. In a random sub-sample (*n* = 251), we also conducted 24-hour dietary recalls (24HDRs) in face-to-face interviews to collect information about recipes, foods that are representative for specific food groups, and site-specific portion sizes. For the translation of food consumption (g/day) into energy (kcal/day) and nutrients intakes (g/day, mg/day, µg/day), Ghana-FPQ data were linked with the latest versions of the West African Food Composition Table [17] and the German Nutrient Database (BLS 3.01, 2010) [18].

### Food variety, dietary diversity, and dietary patterns

In the present study, between-food group variety refers to the number of different food groups that are consumed on a weekly basis. The Food Variety Score (FVS) reflected this concept and was defined as the sum of all foods in pre-defined food groups that are usually consumed per week [9, 19]. For the construction of this count measure, food items of the Ghana-FPQ were collapsed into 20 food groups according to the United Nation’s Food and Agricultural Organization (FAO) food group classification guidance, which consider the similarities in nutrient profiles [20]. The maximum achievable score points for the FVS was 20, and scoring criteria are presented in Table 1.

**Table 1:** Food groups for the construction of the Food Variety Score (FVS) and food categories for the construction of the Dietary Diversity Score (DDS)

No. FVS	FVS food group	Scoring criteria (0–20 points)	No. DDS	DDS food category	Scoring criteria (0–7 points)
1	Starchy roots, tubers, and plantain	At least once per week (0 vs. 1 point)	1	Staples	At least once per day each food group of the category (0 vs. 1 point)
2	Fermented maize products				
3	Bread and cereals				
4	Rice and pasta				
5	Dairy products		2	Dairy	
6	Fish and seafood		3	Meat and alternatives (protein)	
7	Red meat, incl. offals				
8	Processed meat				
9	Poultry				
10	Eggs				
11	Legumes				
12	Nuts and seeds		4	Fruits	
13	Fruits				
14	Vegetables		5	Vegetables	
15	Fats and oils		6	Fats and oils	
16	Sweets				
17	Cakes and cookies				
18	Soft drinks and juices		7	Beverages	
19	Coffee and tea				
20	Alcoholic beverages				

In addition, within-food group variety describes the daily diversification of broader food categories. The corresponding Dietary Diversity Score (DDS) was calculated as the sum of seven food categories, of which the composing food groups were consumed at least once per day [11, 21], i.e., staples, dairy, meat and alternatives (protein), fruits, vegetables, fats and oils, and beverages. Table 1 lists the food groups of the FVS that contributed to DDS food categories and the respective scoring criteria. Accordingly, the maximum achievable score points for the DDS were 7.

The DQI-I was developed “for global monitoring and exploration of diet quality across countries” by Kim et al. [22]. This index assists to describe the diet quality based on food frequencies and nutrient intakes by four major components: variety, adequacy, moderation, and balance. In brief, the DQI-I is based on dietary guidelines and a priori-defined dietary indices. Online Resource 1 presents the components and sub-components of the DQI-I, the maximum achievable score points, and the scoring criteria. Ghana-FPQ data were used to rank participants according to their food and nutrient intakes. The estimation of individual intakes by this semi-quantitative tool was however imprecise. Thus, the present study concentrated on the DQI-I variety component as a measure of between-food group and within-food group varieties (20 score points). The components for adequate intakes of healthy foods and essential nutrients (= adequacy; 40 score points), for foods that should be consumed in moderation (= moderation; 30 score points), and for the overall balance of energy-delivering nutrients and composition of dietary fat (= balance; 10 score points) were used for adjustment in subsequent regression analyses.

Details of the identification of dietary patterns in the RODAM study have been described by Galbete et al. 2017 [15]. Briefly, 134 food items of the Ghana-FPQ were collapsed into 30 food groups, according to their similarities in nutrient composition and culinary use. Exploratory DPs were derived by Principal Component Analysis (PCA) with an orthogonal rotation (VARIMAX), identifying underlying pattern scores that explained the maximum in variance of these 30 food items. Every participant was assigned a pattern score for each dietary pattern to be ranked according to the degree of pattern adherence [15].

### Assessment of covariates

All participants underwent an anthropometric examination in light clothing and without shoes, including weight (kg), height (cm), and waist circumference (cm). Body mass index (BMI) was calculated as weight/(height)^2^ (kg/m^2^). Socio-demographic and lifestyle factors were recorded. These comprised age (years), sex (male and female), and educational status (never been to school or elementary school, lower vocational schooling or lower secondary schooling, intermediate vocational schooling or intermediate/higher secondary schooling, and higher vocational schooling or university). Physical activity was assessed using the World Health Organization (WHO) STEPwise approach to chronic disease risk factor Surveillance (STEPS) questionnaire [23] and was categorized as high, moderate, or low. Smoking status was recorded as current, former or non-smokers.

### Statistical analysis

General characteristics of the RODAM study population are presented as mean ± standard deviation (SD) for normally distributed continuous variables or as median (interquartile range; IQR) for non-normally distributed variables. Between-group comparisons by study site and sex were performed by *t*-test for normally distributed variables and by the non-parametric Wilcoxon rank-sum test for non-normally distributed variables. Categorical data are presented as percentage and were compared between groups using *χ* ^2^-test or Fisher’s exact test.

The relationships of the FVS, the DDS, and the DQI-I variety component with exploratory DPs were examined. Partial Spearman correlations were calculated for the constructed scores (FVS, DDS, and DQI-I) and the previously identified patterns, adjusted for age (years), sex (m/f), study site (5 sites), education (4 categories), energy intake (kcal/day), smoking status (current or former/non-smoker), physical activity (MET-h/week), BMI (kg/m^2^), and waist circumference (cm).

Odds ratios, 95% confidence intervals (CIs), and *p* values for T2D were calculated per 1 score point and per 1 SD of the constructed variety scores using logistic regression. Three models were constructed: model 1 adjusted for age, sex, and study site; model 2: model 1 + education, energy intake, smoking status, and physical activity; and model 3 accounted for an effect of diet on T2D independent of body composition: model 2 + BMI and waist circumference. Participants who knew that they had T2D might have changed their diet because of the diagnosis. To limit this potential of reverse causation, we calculated the associations with T2D among individuals with screen-detected T2D, i.e., excluding self-reported T2D (*n* = 3733). Moreover, replication analyses were performed in an independent study population from urban Ghana (*n* = 1221) [24]. The associations of the FVS, the DDS, and the DQI-I variety component with T2D were compared with those seen in the urban Ghanaian RODAM study site (*n* = 1364) (Online Resource 2).

The associations of DPs with T2D were calculated for each quintile of the pattern scores, using the first quintile as the reference category. Also, for linear associations, we calculated ORs and corresponding 95% CIs for T2D per 1 SD increase of the pattern scores [15]. Finally, the FVS, the DDS, and the DQI-I variety component were included in the multiple-adjusted regression models, relating DPs with T2D, to examine a change in estimate.

## Results

### Study population

The general and basic dietary characteristics of the RODAM study population according to sex and study site are presented in Online Resource 3. RODAM participants were mainly female (63%) and middle-aged (mean age, 46.2 ± 11.1 years). The crude prevalence of T2D was 10%. Men were older, had a higher educational status, had lower BMI and waist circumference, were more likely to be former or current smokers and to consume alcoholic beverages, and were more physically active than women. RODAM participants in Europe had the highest level of education, were more frequently former or current smokers, consumed more alcoholic beverages, and presented with higher BMI and waist circumference than their counterparts in Ghana. The mean length of stay in Europe was 16.9 ± 9.9 years. RODAM participants in rural Ghana had the lowest level of education, the lowest BMI and waist circumference, and were physically more active than those in urban Ghana and Europe. With respect to diet, mean energy intake was higher in men than in women. The highest energy consumption was observed for Europe, followed by rural Ghana and urban Ghana. Carbohydrates, total fat, and protein contributed 53%, 32%, and 14% to the daily energy intake, respectively. This was similar between men and women, but was distinct across study sites: In Europe, energy percentages were shifted towards protein and total fat; in urban Ghana, carbohydrates supplied most of the daily energy; and in rural Ghana, energy intake from carbohydrates was even more pronounced.

### Food variety, dietary diversity, and dietary patterns

The distributions of the FVS, the DDS, and the DQI-I variety component for the total study population and according to study site are presented in Table 2 and in the Online Resource 4. For the total study population, the mean FVS was 12.4 (standard error of the mean (SEM): 0.05), the mean DDS was 5.9 (SEM: 0.02), and the mean score of the DQI-I variety component was 15.7 (SEM: 0.07). All constructed scores were highest in Europe, followed by urban Ghana and rural Ghana (Table 2). We observed moderate positive correlations of the FVS with the DDS (*r* = 0.47; *p* < 0.0001) and also with the DQI-I variety component (*r* = 0.48; *p* < 0.0001). The correlation of the DDS with the DQI-I variety component was *r* = 0.46; *p* < 0.0001.

**Table 2:** Distribution of the Food Variety Score (FVS), the Dietary Diversity Score (DDS), and the variety component with sub-components of the Diet Quality Index-International (DQI-I)

Variety score and score components	Total	Rural Ghana	Urban Ghana	Europe
*N*	3810	870	1364	1576
Food Variety Score (FVS)	12.4 ± 0.05	10.9 ± 0.09	12.3 ± 0.08*	13.4 ± 0.07*
Dietary Diversity Score (DDS)	5.9 ± 0.02	5.3 ± 0.03	5.6 ± 0.04*	6.3 ± 0.02*
Diet Quality Index-International (DQI-I) Variety	15.7 ± 0.07	15.1 ± 0.14	15.6 ± 0.11*	16.0 ± 0.10*
DQI-I overall food group variety (meat/poultry/fish/eggs; dairy/beans; grain; fruit; vegetable)				
≥ 1 serving/day from each food group = 15	38.1 (36.6, 39.7)	37.5 (34.3, 40.7)	40.3 (37.7, 42.9)	36.6 (34.2, 39.0)
Any one food group missing/day = 12	38.1 (36.5, 39.6)	41.2 (37.9, 44.4)	35.0 (32.5, 37.6)	39.0 (36.6, 41.4)
Any two food groups missing/day = 9	17.7 (16.5, 18.9)	16.3 (13.9, 18.8)	16.7 (14.7, 18.7)	19.3 (17.3, 21.2)
Any three food groups missing/day = 6	5.5 (4.8, 6.3)	4.3 (2.9, 5.6)	7.5 (6.1, 8.9)	4.6 (3.5, 5.6)
≥ 4 food groups missing/day = 3	0.6 (0.3, 0.8)	0.8 (0.2, 1.4)	0.5 (0.1, 0.9)	0.5 (0.2, 0.9)
None from any food groups = 0	0.0 (0.0, 0.0)	0.0 (0.0, 0.0)	0.0 (0.0, 0.0)	0.0 (0.0, 0.0)
DQI-I within-food group variety for protein source (meat, poultry, fish, dairy, beans, eggs)				
≥ 3 different sources/day = 5	64.9 (63.3, 66.4)	50.5 (47.1, 53.8)	63.9 (61.4, 66.5)	73.6 (71.4, 75.8)
Two different sources/day = 3	2.6 (2.1, 3.1)	3.0 (1.9, 4.1)	2.7 (1.9, 3.6)	2.3 (1.6, 3.0)
One source/day = 1	12.7 (11.6, 13.7)	17.4 (14.8, 19.9)	14.6 (12.7, 16.5)	8.4 (7.0, 9.7)
None = 0	19.9 (18.6, 21.2)	29.2 (26.2, 32.2)	18.8 (16.7, 20.8)	15.7 (13.9, 17.5)

Data are presented as mean ± standard error of the mean or as percentages (95% confidence interval) of the study participants who fulfilled the scoring criteria. Comparisons with rural Ghana were made by Wilcoxon rank-sum test (* *p* < 0.01)

**Fig. 2:**
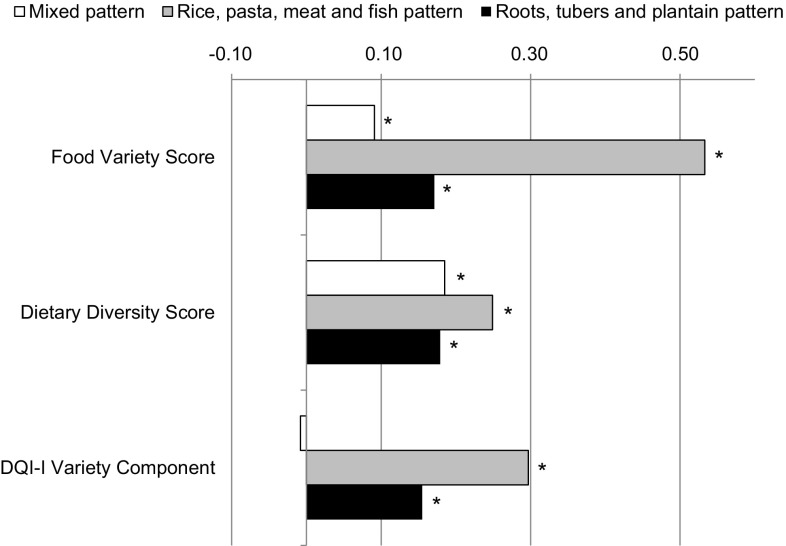
Partial Spearman correlations of the Food Variety Score (FVS), the Dietary Diversity Score (DDS), and the Diet Quality Index-International (DQI-I) variety component with exploratory dietary pattern scores. Correlations were adjusted for age, sex, study site (categorical), education (4 categories), energy intake (kcal/day), smoking (yes/no), physical activity (METs-hour/week), body mass index (kg/m^2^), and waist circumference (cm). Correlations with the DQI-I variety component were additionally adjusted for the other DQI-I components (adequacy, moderation, and balance). Asterisks indicate statistical significance (*p* < 0.001)

Previously, we identified three exploratory DPs, explaining 29% of the total variance in food consumption. Characteristics of these DPs have been reported elsewhere [15]. For the relationships of the variety scores with the identified DPs, partial Spearman correlations were calculated (Fig. 2). All exploratory patterns correlated positively with variety scores. The “mixed” DP was characterized by moderate correlations with the DDS, followed by the FVS and the DQI-I variety component. For the “rice, pasta, meat and fish” DP, we observed a strong correlation with the FVS, and moderate relationships with the DQI-I variety component and the DDS. The “roots, tubers, and plantain” DP correlated poorly with variety scores (each, *r* < 0.18). These relationships were similar across study sites (Table 3).

**Table 3:** Partial Spearman correlations of the Food Variety Score (FVS), the Dietary Diversity Score (DDS), and the variety component of the Diet Quality Index-International (DQI-I) with exploratory dietary pattern scores, according to RODAM study site

Correlation coefficients	Mixed dietary pattern			Rice, pasta, meat and fish dietary pattern			Roots, tubers and plantain dietary pattern		
	Rural Ghana	Urban Ghana	Europe	Rural Ghana	Urban Ghana	Europe	Rural Ghana	Urban Ghana	Europe
FVS	**0.19**	**0.16**	**0.26**	**0.58**	**0.58**	**0.43**	0.06	**0.19**	**0.17**
DDS	**0.18**	**0.21**	**0.30**	**0.30**	**0.24**	**0.19**	−0.01	**0.23**	**0.20**
DQI-I variety	−0.01	0.02	**0.12**	**0.31**	**0.28**	**0.24**	**0.15**	**0.16**	**0.11**

Correlations were adjusted for age, sex, education (4 categories), energy intake (kcal/day), smoking (yes/no), physical activity (METs-h/week), Body Mass Index (kg/m^2^), and waist circumference (cm). Correlations with the DQI-I variety component were additionally adjusted for the other DQI-I components (adequacy, moderation, balance). Figures in bold represent significant correlations (*p* < 0.0001)

### Food variety, dietary diversity, and type 2 diabetes

The associations of the FVS, the DDS, and the DQI-I variety component with T2D are presented in Table 4. The FVS was inversely associated with T2D, adjusted for age, sex, and study site. This effect remained after adjustment for educational attainment, energy intake, smoking status, physical activity, BMI, and waist circumference (OR per 1 score-SD increase: 0.81; 95% CI 0.71, 0.93). No significant relationships were seen for the DDS and the DQI-I variety component with T2D. There were no interactions for the identified associations with study site (Table 4).

**Table 4:** Associations of the Food Variety Score (FVS), the Dietary Diversity Score (DDS), and the variety component of the Diet Quality Index-International (DQI-I) with type 2 diabetes among 3810 Ghanaian adults

Dietary score	Per 1 score point		Per 1 standard deviation of the score		*p* for interaction with study site
	OR (95% CI)	*p*	OR (95% CI)	*p*	
Food Variety Score (FVS)					
Model 1	0.92 (0.88, 0.96)	0.0001	0.79 (0.70, 0.89)	0.0001	0.560
Model 2	0.94 (0.89, 0.98)	0.006	0.83 (0.72, 0.95)	0.006	0.450
Model 3	0.93 (0.89, 0.98)	0.003	0.81 (0.71, 0.93)	0.003	0.318
Dietary Diversity Score (DDS)					
Model 1	1.00 (0.89, 1.11)	0.929	1.00 (0.88, 1.12)	0.929	0.939
Model 2	1.05 (0.94, 1.18)	0.396	1.06 (0.93, 1.20)	0.396	0.828
Model 3	1.05 (0.94, 1.18)	0.420	1.06 (0.93, 1.20)	0.420	0.760
DQI-I variety component					
Model 1	1.00 (0.97, 1.03)	0.925	0.99 (0.86, 1.14)	0.925	0.489
Model 2	1.00 (0.97, 1.03)	0.975	1.00 (0.87, 1.15)	0.975	0.497
Model 3	1.00 (0.96, 1.03)	0.874	0.99 (0.86, 1.14)	0.874	0.389

Odds ratios (ORs), 95% confidence intervals (CIs), and *p* values were calculated by logistic regression; the significance of the cross-product term with study site was evaluated (*p* for interaction). Model 1: adjusted for age, sex, study site (categorical); model 2: model 1 + education (4 categories), energy intake (kcal/day), smoking (yes/no), physical activity (METs-h/week); model 3: model 2 + Body Mass Index (kg/m^2^) and waist circumference (cm). The DQI-I variety component was additionally adjusted for the other DQI-I components (adequacy, moderation, and balance)

In a sensitivity analysis for screen-detected T2D, i.e., excluding self-reported T2D (*n* = 3733), the multiple-adjusted associations per 1 score-SD increase were similar (FVS: 0.81; 95% CI 0.69, 0.96; DDS: 1.10; 95% CI 0.94, 1.28; DQI-I variety component: 1.07; 95% CI 0.90, 1.28). In addition, in an independent urban Ghanaian study population (*n* = 1221), we also observed an inverse association of the FVS with T2D (OR per 1 score-SD increase: 0.63; 95% CI 0.54, 0.73). Again, in this replication study, there were no associations for the DDS and the DQI-I variety component (ESM 2).

For associations of previously identified DPs with T2D, the results are presented in Table 5. Per 1 score-SD of the “rice, pasta, meat and fish” DP, the odds of T2D decreased by 18% in the fully adjusted model (OR per 1 score-SD increase: 0.82; 95% CI 0.71, 0.95). This effect attenuated after adjustment for the FVS to OR: 0.89; 95% CI 0.75, 1.06, whereas neither the DDS nor the DQI-I variety component influenced the association between the “rice, pasta, meat and fish” DP and T2D. The “mixed” DP and the “roots, tubers, and plantain” DP were not associated with T2D, and this was still discernible after adjustments for the FVS, the DDS or the DQI-I variety component (Table 5).

**Table 5:** Multiple-adjusted associations of exploratory dietary patterns with type 2 diabetes and the changes in effect by the Food Variety Score (FVS), the Dietary Diversity Score (DDS), and the variety component of the Diet Quality Index-International (DQI-I)

Model	Odds ratio (95% confidence interval)											
	Q1	Q2		Q3		Q4		Q5		*p* trend	Per 1 score-SD	
Mixed dietary pattern												
Diabetes/controls	70/690	59/690		54/691		89/690		87/690				
Adjusted model	1.00	0.79	0.54–1.16	0.76	0.50–1.15	0.89	0.49–1.61	0.84	0.44–1.62	0.601	1.00	0.81–1.23
+ FVS	1.00	0.82	0.56–1.20	0.81	0.54–1.23	0.97	0.54–1.77	0.96	0.50–1.86	0.671	1.04	0.84–1.28
+ DDS	1.00	0.79	0.54–1.16	0.74	0.49–1.12	0.85	0.47–1.56	0.80	0.41–1.55	0.481	0.98	0.79–1.21
+ DQI-I variety	1.00	0.75	0.51–1.10	0.70	0.46–1.06	0.78	0.42–1.44	0.71	0.36–1.40	0.383	0.95	0.76–1.19
Rice, pasta, meat and fish pattern												
Diabetes/controls	107/690	90/690		62/691		58/690		42/690				
Adjusted model	1.00	0.92	0.67–1.27	0.64	0.44–0.91	0.67	0.46–0.98	0.59	0.38–0.91	0.014	0.82	0.71–0.95
+ FVS	1.00	0.97	0.70–1.35	0.70	0.48–1.02	0.77	0.51–1.17	0.71	0.43–1.16	0.208	0.89	0.75–1.06
+ DDS	1.00	0.91	0.66–1.25	0.61	0.43–0.88	0.64	0.43–0.93	0.55	0.35–0.85	0.005	0.80	0.68–0.93
+ DQI-I variety	1.00	0.81	0.59–1.13	0.54	0.37–0.77	0.51	0.34–0.76	0.42	0.25–0.69	0.012	0.81	0.68–0.97
Roots, tubers and plantain pattern												
Diabetes/controls	83/690	93/690		71/691		62/690		50/690				
Adjusted model	1.00	1.25	0.89–1.76	1.01	0.70–1.46	1.05	0.71–1.56	1.15	0.73–1.80	0.944	1.06	0.90–1.24
+ FVS	1.00	1.29	0.92–1.82	1.09	0.75–1.58	1.18	0.79–1.77	1.27	0.80–1.99	0.631	1.10	0.94–1.29
+ DDS	1.00	1.24	0.88–1.75	0.99	0.68–1.43	1.01	0.67–1.52	1.11	0.70–1.75	0.927	1.04	0.89–1.23
+ DQI-I variety	1.00	1.29	0.92–1.81	1.00	0.68–1.47	1.07	0.70–1.63	1.13	0.70–1.83	0.865	1.02	0.86–1.21

Odds ratios (ORs), 95% confidence intervals (CIs), and *p* values were calculated by logistic regression and were adjusted for age, sex, study site (categorical), education (4 categories), energy intake (kcal/day), smoking (yes/no), physical activity (METs-h/week), Body Mass Index (kg/m^2^), and waist circumference (cm). The trend test across quintiles (Q) was calculated by modeling the medians of the DP score quintiles as a continuous variable (*p* trend). Models with the DQI-I variety component were additionally adjusted for the other DQI-I components (adequacy, moderation, and balance)

## Discussion

### Summary of main results

The current study investigated the effects of dietary diversification and previously identified DPs on T2D risk in a large multi-center cross-sectional study among Ghanaian adults. Scores for dietary diversification correlated positively with DPs, most strongly with the “rice, pasta, meat and fish” DP. In the multiple-adjusted linear model, the FVS (per 1 score-SD increase) reduced the odds of T2D by 19% (Table 4). This effect was not modified by study site (Table 4) and was confirmed in an independent urban Ghanaian study population (Table ESM 2.1). The FVS attenuated the inverse association between a “rice, pasta, meat and fish” pattern with T2D from 18% to 11% (Table 5). Further, no associations with T2D were seen for the DDS and the DQI-I variety component, and this was also true for another two exploratory DPs.

### Food variety, dietary diversity, and dietary patterns

In this adult Ghanaian population, the “roots, tubers and plantain” DP, which dominated in rural Ghana, showed the weakest correlations with between- and within-food group varieties. Poor dietary diversification of this DP might stem from seasonal influences on food availability as well as reduced biodiversity, because of changes in agriculture and trade [25]. In addition, low household SES and long market distances in rural areas of sub-Saharan Africa limit the access to different foods [26–28]. In contrast, the DPs that prevailed in urban Ghana and Europe were characterized by a mixture of fresh foods and manufactured groceries (“mixed” DP; “rice, pasta, meat and fish” DP), which showed good between- and within-food group varieties. Indeed, diversification was seen for both, Ghana-specific food items and products of the European food markets. The constant availability of a variety of foods in large African cities and in Europe [29] might be responsible for these findings. Moreover, participants with higher adherence to the “mixed” and the “rice, pasta, meat and fish” DPs had a higher SES, which likely improves food access.

### Food variety, dietary diversity, and type 2 diabetes

The present description of the importance of food variety and dietary diversity for T2D in sub-Saharan African populations extends the findings about dietary diversification and T2D in other ethnic groups [30]. The first epidemiologic evidence for a health-beneficial effect of diversification indices was reported from Swedish and US American cohorts, where food variety and dietary diversity reduced all-cause and cause-specific mortality [21, 31, 32]. Recently, an analysis from the European Prospective Investigation into Cancer and Nutrition (EPIC)-Norfolk study showed that greater total dietary diversity was associated with 30% lower risk of developing T2D, comparing diets of the five major food groups [dairy products, fruits, vegetables, grain/cereal products, and meat and alternatives (protein)] with three or fewer food groups [33]. The inverse association of the FVS (between-food group variety) with T2D observed at all RODAM study sites agrees with these health-beneficial effects. Yet, the biological mechanisms behind dietary diversification and reduced T2D risk remain to be elucidated. Experimental evidence suggests that a greater diversity of foods influences the composition of gut microbiota, thereby improving immune function and health outcomes [34]. Moreover, dietary diversification may increase the intakes of micronutrients, dietary fiber, and secondary plant metabolites, such as flavonoids and carotenoids, which are known to have health-beneficial effects [35, 36].

The null associations of the DDS and the DQI-I variety component with T2D in the RODAM study contrast the protective effect of within-food group variety in EPIC-Norfolk [33], but are in line with the lack of association between dietary diversity and T2D in the Multi-Ethnic Study of Atherosclerosis (MESA) cohort [37]. The authors of the latter concluded that there is “little evidence for the benefits of diet diversity for diabetes”. From a mechanistic perspective, greater within-food group variety may increase both, the intakes of healthy and unhealthy foods [37]. In fact, in developing regions and in population-dense countries, the (sudden) increase in food production may lead to a loss in diet quality [25]. As a consequence, and possibly explaining parts of the absent associations in our study, the adverse effects of increased diversity for animal-based products and sugar-sweetened beverages, which contain high amounts of total fat, trans-fats, sodium, and simple carbohydrates, might override the benefit of greater fruit and vegetable diversity.

### Strengths and limitations

To the best of our knowledge, this study provides first evidence on the role of dietary diversification for the risk of T2D among sub-Saharan African individuals living in their country of origin and in three large European cities. Nevertheless, our findings need careful interpretation. While the Ghana-FPQ was pre-tested at all study sites [15], this cultural sensitive instrument remains to be validated. Generally, food frequency questionnaires (FFQs) tend to overestimate or underestimate food intake [38], which might have diluted the observed relationships with T2D. Similarly, the diet-disease associations could be affected by the retrospective dietary assessment, because of the inherent risk of recall bias. On the other hand, FFQs constitute excellent instruments to depict the long-term dietary behavior in cross-sectional studies and can rank the participants according to their food intake [38]. The present study design did not allow for time sequence analysis, and reverse causation cannot be excluded among individuals with previously diagnosed T2D. Yet, similar results were obtained when the analysis was restricted to individuals with screen-detected T2D. Reassuringly, the results for RODAM urban Ghana were independently replicated among urban Ghanaian adults. While unmeasured and residual confounding might have distorted our findings, we have accounted for total energy intake and other important confounders in the present study.

## Conclusions

In conclusion, among this Ghanaian study population, increased variety between-food groups might exert beneficial effects on glucose metabolism and contribute to the inverse association of the “rice, pasta, meat and fish” DP with T2D. Yet, diversification within food groups may be less relevant for T2D risk in this population group. Longitudinal studies will be required to verify the observed relationships of dietary diversification and exploratory DPs with T2D among populations of sub-Saharan African origin.

## Electronic supplementary material

Below is the link to the electronic supplementary material.

Supplementary material 1 (DOCX 36 kb)
