# Supplementary material for: Food variety, dietary diversity, and type 2 diabetes in a multi-center cross-sectional study among Ghanaian migrants in Europe and their compatriots in Ghana: the RODAM study
Source: Eur J Nutr. 2017 Sep 25;57(8):2723–33. doi: 10.1007/s00394-017-1538-4 (PMC6267387; doi:10.1007/s00394-017-1538-4)
Supplement: Supplementary file 1 — Supplementary material 1 (DOCX 36 kb) [file 394_2017_1538_MOESM1_ESM.docx]

**Title:** Food variety, dietary diversity and type 2 diabetes in a multi-center cross-sectional study among Ghanaian migrants in Europe and their compatriots in Ghana: The RODAM Study

**Journal:** European Journal of Nutrition

Ina Danquah, Cecilia Galbete, Karlijn Meeks, Mary Nicolaou, Kerstin Klipstein-Grobusch, Juliet Addo, Ama de-Graft Aikins, Stephen K. Amoah, Peter Agyei-Baffour, Joachim Spranger^j^, Liam Smeeth, Ellis Owusu-Dabo, Charles Agyemang, Frank P. Mockenhaupt, Erik Beune, Matthias B. Schulze

**Corresponding author:**

Dr. Ina Danquah

Department of Molecular Epidemiology

German Institute of Human Nutrition Potsdam-Rehbruecke (DIfE)

Arthur-Scheunert-Allee 114-116

14558 Nuthetal, Germany

Email: ina.danquah@dife.de

Phone: +49(0)33200 882453

Fax: +49(0)33200 882477

Online Resource 1: Scoring criteria for the components and sub-components of the Diet Quality Index-International

| **Component** | **Scoring criteria** |
| --- | --- |
| Variety component |  |
| Overall food group variety (meat/poultry/fish/eggs; dairy/beans; grain; fruit; vegetable) | ≥1 serving/d from each food group = 15  Any 1 food group missing/d = 12  Any 2 food groups missing/d = 9  Any 3 food groups missing/d = 6  ≥4 food groups missing/d = 3  None from any food groups = 0 |
| Within-group variety for protein source (meat, poultry, fish, dairy, beans, eggs) | ≥3 different sources/d = 5  2 different sources/d = 3  1 source/d = 1  None = 0 |
| Adequacy component |  |
| Vegetable group | ≥3-5 times/d = 5; 0 times/d = 0  ≥100%  <100-50%  <50% |
| Fruit group | ≥2-4 times/d = 5; 0 times/d = 0  ≥100%  <100-50%  <50% |
| Grain group | ≥6-11 times/d = 5; 0 times/d = 0  ≥100%  <100-50%  <50% |
| Fiber | ≥20-30 g/d = 5; 0 g/d = 0  ≥100%  <100-50%  <50% |
| Protein | ≥10% energy/d = 5; 0% energy/d = 0  ≥100%  <100-50%  <50% |
| Iron | ≥100% RDA = 5; 0% RDA = 0  ≥100%  <100-50%  <50% |
| Calcium | ≥100% AI = 5; 0% AI = 0  ≥100%  <100-50%  <50% |
| Vitamin C | ≥100% RDA = 5; 0% RDA = 0  ≥100%  <100-50%  <50% |

**ESM 1: continued**

| **Component** | **Scoring criteria** |
| --- | --- |
| Moderation component |  |
| Total fat | ≤20% energy/d = 6  >20-30% energy/d = 3  >30% energy/d = 0 |
| Saturated fat | ≤7% energy/d = 6  >7-10% energy/d = 3  >10% energy/d = 0 |
| Cholesterol | ≤300 mg/d = 6  >300-400 mg/d = 3  >400 mg/d = 0 |
| Sodium | ≤2400 mg/d = 6  >2400-3400 mg/d = 3  >3400 mg/d = 0 |
| Empty calorie foods* | ≤3% energy/d = 6  >3-10% energy/d = 3  >10% energy/d = 0 |
| Balance component |  |
| Macronutrient ratio  (carbohydrate:protein:fat) | 55-65:10-15:15-25 = 6  52-68:9-16:13-27 = 4  50-70:8-17:12-30 = 2  otherwise = 0 |
| Fatty acid ratio  (PUFA:MUFA:SFA) | P/S=1-1.5 and M/S=1-1.5 = 4  P/S=0.8-1.7 and M/S=0.8-1.7 = 2  otherwise = 0 |

AI, Adequate Intakes; RDA; Recommended Dietary Allowance [39, 40]; MUFA, monounsaturated fatty acids; M/S, ratio of MUFA to SFA intake; PUFA; polyunsaturated fatty acids; P/S; ratio of PUFA to SFA intake; SFA, saturated fatty acids

*, Empty calorie foods were defined as food items that contain energy ≥300 kcal/100 g edible food *plus* fiber <500 mg/100 g edible food or minerals <500 mg/100 g edible food.

**Online Resource 2: External replication using data of the Kumasi Diabetes and Hypertension (KDH) study**

The KDH study was conducted as an unmatched hospital-based case-control study in Kumasi, Ghana from August 2007 through June 2008.[24] The primary aim of the study was to identify genetic and non-genetic risk factors for T2D and hypertension in urban Ghana (n = 1,466). Cases were recruited at the diabetes center and the hypertension clinic, Komfo Anokye Teaching Hospital. Preliminary controls were recruited among their friends, neighbors and community members, among outpatient attendants, and among hospital staff. After a 10-hours overnight fast, a venous blood sample was drawn from each participant for measurements of fasting plasma glucose (FPG; mmol/L). Type 2 diabetes (T2D) was defined as FPG ≥7.0 mmol/L or documented anti-diabetic medication. Dietary data, demographic, socio-economic, and medical history information were documented in questionnaire-based interviews. For the assessment of the usual daily food intake, a Food Frequency Questionnaire (FFQ) documented the weekly consumption of 51 food items in ten food categories over the past 12 months covering six response categories. Further, single 24-Hours Dietary Recalls (24HDRs) were applied to record the types and amounts of consumed foods of the preceding 24 hours. Food consumption data were linked with nutrient composition data (West African Food Composition Database 2012[17] to translate the amount of consumed foods (g/d) into energy (kcal/d) and nutrient intakes (g/d, mg/d or µg/d). Demographic, socio-economic and medical history data comprised age (years), sex, education (none, primary, secondary, tertiary or other), occupation (subsistence farmer, commercial farmer, casual labourer, artisan, trader, businessman/woman, public servant, unemployed or other), own and family history of diabetes (yes or no), and smoking status (never, quit or current). Daily energy expenditure (kJ/d) was calculated as the sum of metabolic equivalents corresponding to activity intensities (METs).[41] Anthropometric measures were taken; body mass index (BMI) was calculated as weight/(height)^2^ (kg/m^2^), and waist-to-hip ratio (WHR) as waist circumference/hip circumference. For the present analysis, the final analytical sample size was 1,221, after the exclusion of 245 participants with missing or implausible data on dietary habits, anthropometry, and socio-economic status.

For the construction of the FVS and the DDS, food items of the FFQ were collapsed into 20 food groups according to the United Nation’s Food and Agricultural Organization (FAO) food group classification guidance, which considers the similarities in nutrient profiles.[20] The scores were constructed as described in **Table 1**. For the DQI-I, FFQ data were used to construct components and sub-components relating to food groups (variety, adequacy), while 24HDR data were used to calculate nutrients-based components and sub-components (adequacy, moderation, balance; **ESM 1**).

For the associations with T2D, odds ratios, 95% confidence intervals and *p*-values were calculated per 1 standard deviation of the scores by logistic regression. The regression models were adjusted for age (years), sex, family history of diabetes (yes/no), unemployment (yes/no), education (4 categories), literacy (able/unable), smoking (quit or current/never), energy expenditure (kcal/d), body mass index (kg/m^2^), and waist-to-hip ratio. Models with the DQI-I variety component were additionally adjusted for the other DQI-I components (adequacy, moderation, balance).

**Table ESM 2.1: Associations of the Food Variety Score (FVS), the Dietary Diversity Score (DDS), the Diet Quality Index-International (DQI-I) variety component, and dietary patterns with type 2 diabetes in urban Ghanaian adults**

| **Dietary scores** | **Odds ratio per 1 standard deviation of the score (95% CI) and *p*-value** | | | |
| --- | --- | --- | --- | --- |
|  | **RODAM study (n = 1,364)** | | **KDH study (n = 1,221)** | |
| **Food Variety Score** | 0.73 (0.57, 0.92) | 0.008 | 0.63 (0.54, 0.73) | <.0001 |
| **Dietary Diversity Score** | 1.16 (0.97, 1.38) | 0.114 | 0.91 (0.79, 1.05) | 0.212 |
| **Diet Quality Index-International, variety component** | 0.98 (0.77, 1.25) | 0.857 | 1.04 (0.89, 1.22) | 0.593 |

Odds ratios, 95% confidence intervals (CIs) and *p*-values were calculated by logistic regression. For the Research on Obesity and Diabetes among African Migrants (RODAM) study, the models were adjusted for age (years), sex, study site (5 categories), education (4 categories), energy intake (kcal/d), smoking (current or previous/never), physical activity (METs-h/week), body mass index (kg/m^2^) and waist circumference (cm). For the Kumasi Diabetes and Hypertension (KDH) study, the models were adjusted for age (years), sex, family history of diabetes (yes/no), unemployment (yes/no), education (4 categories), literacy (able/unable), smoking (quit or current/never), energy expenditure (kcal/d), body mass index (kg/m^2^), and waist-to-hip ratio. Models with the DQI-I variety component were additionally adjusted for the other DQI-I components (adequacy, moderation, balance).

Online Resource 3: General characteristics of the RODAM study population by gender and study site

| **Characteristics** | **Total**  **(n = 3,810)** | **Men**  **(n = 1,407)** | **Women**  **(n = 2,403)** | **Rural Ghana**  **(n = 870)** | **Urban Ghana**  **(n = 1,364)** | **Europe**  **(n = 1,576)** |
| --- | --- | --- | --- | --- | --- | --- |
| Age (years) | 46.2 ± 11.1 | 47.0 ± 11.4 | 45.7 ± 10.9 | 46.7 ± 12.6 | 45.3 ± 11.4 | 46.6 ± 9.8 |
| Sex (female) | 63.1 | - | - | 61.3 | 72.2 | 56.1 |
| Education |  |  |  |  |  |  |
| Never or elementary | 37.9 | 22.3 | 47.0 | 57.4 | 44.0 | 21.9 |
| Low | 37.4 | 41.4 | 35.1 | 31.3 | 38.8 | 39.7 |
| Intermediate | 16.1 | 22.2 | 12.5 | 7.9 | 12.5 | 23.7 |
| Higher vocational | 8.6 | 14.1 | 5.4 | 3.6 | 4.8 | 14.7 |
| Body mass index (kg/m^2^) | 26.7 ± 5.5 | 24.8 ± 4.5 | 27.8 ± 5.7 | 22.6 ± 4.3 | 26.9 ± 5.4 | 28.7 ± 5.0 |
| Waist circumference (cm) | 89. 6 ± 12.6 | 86.9 ± 12.2 | 91.1 ± 12.6 | 81.2 ± 10.9 | 89.4 ± 11.8 | 94.3 ± 11.6 |
| Smoking (current or former) | 9.3 | 19.8 | 3.3 | 8.2 | 6.8 | 12.2 |
| Physical activity (MET-h/week) | 72 (14,168) | 96 (28,198) | 60 (10,156) | 92 (38,166) | 60 (6, 156) | 64 (14,190) |
| Total Energy intake (kcal/day) | 2529 ± 837 | 2619 ± 859 | 2476 ± 820 | 2629 ± 847 | 2297 ± 660 | 2675 ± 922 |
| Carbohydrates intake (energy %) | 53.2 ± 9.1 | 52.5 ± 9.4 | 53.6 ± 8.9 | 56.4 ± 8.3 | 54.5 ± 8.1 | 50.4 ± 9.5 |
| Fat intake (energy %) | 32.3 ± 8.3 | 32.2 ± 8.6 | 32.4 ± 8.1 | 31.4 ± 7.3 | 31.6 ± 7.2 | 33.4 ± 9.4 |
| Protein intake (energy %) | 13.5 ± 2.6 | 13.4 ± 2.6 | 13.5 ± 2.7 | 11.5 ± 2.2 | 13.6 ± 2.5 | 14.4 ± 2.5 |
| Intake of alcoholic beverages (g/d) | 0.1 (0.0, 1.8) | 0.8 (0.0, 5.1) | 0.1 (0.0, 1.1) | 0.1 (0.0, 1.3) | 0.1 (0.0, 0.6) | 0.9 (0.0, 4.8) |

Data are presented in mean ± standard deviation, median (interquartile range) or percentage.

**Online Resource 4: Distribution of the Food Variety Score (FVS) food groups, the Dietary Diversity Score (DDS) food categories and the variety component of the Diet Quality Index-International (DQI-I) subcomponents**

| **Variety score and score components** | **Total** | **Rural Ghana** | **Urban Ghana** | **Europe** |
| --- | --- | --- | --- | --- |
| N | 3,810 | 870 | 1,364 | 1,576 |
| **Food Variety Score (FVS)** | 12.4 ± 0.05 | 10.9 ± 0.09 | 12.3 ± 0.08 * | 13.4 ± 0.07 * |
| Roots, tubers and plantain | 99.0 (98.7, 99.3) | 100 (100, 100) | 99.2 (98.7, 99.7) | 98.2 (97.6, 98.9) |
| Fermented maize products | 68.2 (66.7, 69.7) | 92.0 (90.2, 93.8) | 86.5 (84.7, 88.3) | 39.3 (36.9, 41.7) |
| Bread and cereals | 53.1 (51.5, 54.7) | 52.2 (48.9, 55.5) | 56.2 (53.6, 58.9) | 50.9 (48.4, 53.4) |
| Rice and pasta | 92.7 (91.9, 93.5) | 85.8 (83.4, 88.1) | 96.4 (95.4, 97.4) | 93.3 (92.0, 94.5) |
| Dairy products | 65.8 (64.3, 67.3) | 35.3 (32.1, 38.5) | 62.8 (60.2, 65.3) | 85.3 (83.6, 87.1) |
| Fish and seafood | 91.8 (90.9, 92.7) | 90.3 (88.4, 92.3) | 97.7 (96.9, 98.5) | 87.6 (85.9, 89.2) |
| Red meat | 61.4 (59.8, 62.9) | 51.5 (48.2, 54.8) | 57.9 (55.3, 60.5) | 69.8 (67.5, 72.1) |
| Processed meat | 17.7 (16.5, 18.9) | 3.8 (2.5, 5.1) | 9.1 (7.6, 10.6) | 32.8 (30.5, 35.1) |
| Poultry | 48.5 (46.9, 50.1) | 30.8 (27.7, 33.9) | 32.0 (29.5, 34.4) | 72.6 (70.4, 74.8) |
| Eggs | 36.9 (35.3, 38.4) | 26.7 (23.7, 29.6) | 35.0 (32.5, 37.6) | 44.0 (41.6, 46.5) |
| Legumes | 77.5 (76.2, 78.8) | 76.1 (73.3, 78.9) | 80.3 (78.2, 82.4) | 75.8 (73.7, 77.9) |
| Nuts and seeds | 41.7 (40.1, 43.3) | 36.3 (33.1, 39.5) | 46.9 (44.3, 49.6) | 40.1 (37.7, 42.5) |
| Fruits | 97.2 (96.7, 97.7) | 96.6 (95.3, 97.8) | 97.3 (96.4, 98.2) | 97.5 (96.8, 98.3) |
| Vegetables | 100 (100, 100) | 100 (100, 100) | 100 (100, 100) | 100 (100, 100) |
| Fats and oils | 96.1 (95.5, 96.7) | 94.1 (92.6, 95.7) | 97.8 (97.0, 98.6) | 95.8 (94.8, 96.7) |
| Sweets | 11.2 (10.2, 12.2) | 5.1 (3.6, 6.5) | 9.4 (7.8, 10.9) | 16.1 (14.3, 17.9) |
| Cakes and cookies | 50.1 (48.5, 51.7) | 41.8 (38.6, 45.1) | 57.0 (54.4, 59.7) | 48.6 (46.1, 51.1) |
| Soft drinks and juices | 57.5 (56.0, 59.1) | 26.8 (23.8, 29.7) | 50.6 (47.9, 53.2) | 80.5 (78.6, 82.5) |
| Coffee and tea | 60.5 (58.9, 62.0) | 27.8 (24.8, 30.8) | 51.0 (48.3, 53.6) | 86.7 (85.1, 88.4) |
| Alcoholic beverages | 17.6 (16.4, 18.8) | 13.7 (11.4, 16.0) | 7.7 (6.3, 9.1) | 28.4 (26.1, 30.6) |

**ESM 4: continued**

| **Variety score and score components** | **Total** | **Rural Ghana** | **Urban Ghana** | **Europe** |
| --- | --- | --- | --- | --- |
| N | 3,810 | 870 | 1,364 | 1,576 |
| **Dietary Diversity Score (DDS)** | 5.9 ± 0.02 | 5.3 ± 0.03 | 5.6 ± 0.04 * | 6.3 ± 0.02 * |
| Staples | 99.6 (99.4, 99.8) | 100 (100, 100) | 100 (100, 100) | 99.0 (98.5, 99.5) |
| Dairy | 48.2 (46.7, 49.8) | 21.0 (18.3, 23.7) | 45.4 (42.7, 48.0) | 65.7 (63.4, 68.1) |
| Meat | 97.3 (96.8, 97.8) | 97.4 (96.3, 98.4) | 98.7 (98.1, 99.3) | 96.0 (95.0, 97.0) |
| Fruits | 87.0 (86.0, 88.1) | 92.2 (90.4, 94.0) | 80.7 (78.6, 82.8) | 89.7 (88.2, 91.2) |
| Vegetables | 100 (100, 100) | 100 (100, 100) | 100 (100, 100) | 100 (100, 100) |
| Fats | 81.4 (80.2, 82.7) | 80.3 (77.7, 83.0) | 74.0 (71.7, 76.3) | 88.5 (86.9, 90.1) |
| Beverages | 72.5 (71.1, 73.9) | 44.0 (40.7, 47.3) | 64.9 (62.4, 67.4) | 94.7 (93.6, 95.8) |
| **Diet Quality Index-International (DQI-I)**  **Variety component** | 15.7 ± 0.07 | 15.1 ± 0.14 | 15.6 ± 0.11 * | 16.0 ± 0.10 * |
| Overall food group variety (meat/poultry/fish/eggs; dairy/beans; grain; fruit; vegetable) |  |  |  |  |
| ≥1 serving/d from each food group = 15  Any 1 food group missing/d = 12  Any 2 food groups missing/d = 9  Any3 food groups missing/d = 6  ≥4 food groups missing/d = 3  None from any food groups = 0 | 38.1 (36.6, 39.7)  38.1 (36.5, 39.6)  17.7 (16.5, 18.9)  5.5 (4.8, 6.3)  0.6 (0.3, 0.8)  0.0 (0.0, 0.0) | 37.5 (34.3, 40.7)  41.2 (37.9, 44.4)  16.3 (13.9, 18.8)  4.3 (2.9, 5.6)  0.8 (0.2, 1.4)  0.0 (0.0, 0.0) | 40.3 (37.7, 42.9)  35.0 (32.5, 37.6)  16.7 (14.7, 18.7)  7.5 (6.1, 8.9)  0.5 (0.1, 0.9)  0.0 (0.0, 0.0) | 36.6 (34.2, 39.0)  39.0 (36.6, 41.4)  19.3 (17.3, 21.2)  4.6 (3.5, 5.6)  0.5 (0.2, 0.9)  0.0 (0.0, 0.0) |
| Within-food group variety for protein source  (meat, poultry, fish, dairy, beans, eggs) |  |  |  |  |
| ≥3 different sources/d = 5  2 different sources/d = 3  1 source/d = 1  None = 0 | 64.9 (63.3, 66.4)  2.6 (2.1, 3.1)  12.7 (11.6, 13.7)  19.9 (18.6, 21.2) | 50.5 (47.1, 53.8)  3.0 (1.9, 4.1)  17.4 (14.8, 19.9)  29.2 (26.2, 32.2) | 63.9 (61.4, 66.5)  2.7 (1.9, 3.6)  14.6 (12.7, 16.5)  18.8 (16.7, 20.8) | 73.6 (71.4, 75.8)  2.3 (1.6, 3.0)  8.4 (7.0, 9.7)  15.7 (13.9, 17.5) |

Data are presented as mean ± standard error of the mean or as percentages (95% confidence interval) of the study participants who fulfilled the scoring criteria. Comparisons with rural Ghana were made by Wilcoxon rank-sum test (* *p* < 0.01)
